# Radionuclide Imaging of Dual Ectopic Thyroid in a Preadolescent Girl

**DOI:** 10.4274/mirt.46220

**Published:** 2014-10-05

**Authors:** Şule Yıldırım, Hasan İkbal Atılgan, Meliha Korkmaz, Koray Demirel, Gökhan Koca

**Affiliations:** 1 Dicle University Faculty of Medicine, Department of Nuclear Medicine, Diyarbakır, Turkey; 2 Ankara Training and Research Hospital, Clinic of Nuclear Medicine, Ankara, Turkey

**Keywords:** Thyroid, scintigraphy, congenital hypothyroidism

## Abstract

Ectopic thyroid is a congenital defect in which the thyroid gland is located away from the usual pretracheal location. Dual ectopic thyroid, which consists of two foci of thyroid tissue, is very rare. In this case dual ectopic thyroid with subclinical hypothyroidism in a 10-year-old-girl was reported. The absence of the thyroid gland in the pretracheal location was revealed by ultrasonography (USG). Two foci of ectopic thyroid tissue located at the base of the tongue and infrahyoid region were determined by Technetium-99m pertechnetate thyroid scintigraphy. It can be concluded that if the thyroid gland is not visible by USG, ectopic thyroid tissue should be evaluated with scintigraphy.

## Abstract (tr)

Ektopik tiroid, tiroid bezinin olağan pretrakeal lokalizasyonunun dışında yerleşim gösterdiği konjenital bir defekttir. İki tiroid dokusu odağından oluşan dual ektopik tiroid çok nadirdir. Bu olguda subklinik hipotiroidi ile birlikte dual ektopik tiroidi olan 10 yaşındaki kız çocuğu sunulmuştur. Ultrasonografide (USG) pretrakeal lokalizasyonda tiroid dokusunun olmadığı gösterildi. Teknesyum-99m perteknetat tiroid sintigrafisi ile dil kökünde ve infrahyoid alanda lokalize iki odak halinde ektopik tiroid dokusu saptandı. Sonuç olarak tiroid bezi USG ile görülmediğinde, ektopik tiroid dokusu sintigrafi ile birlikte değerlendirilmelidir.

## INTRODUCTION

Ectopic thyroid is a developmental defect of the thyroid gland, in which the thyroid tissue is in a site other than the pretracheal location. Ectopic thyroid tissues can be seen in many sites from the base of tongue through to the diaphragm (1). It is most commonly presented as lingual thyroid and is the only thyroid in 70% of all cases (2). It is very uncommon to encounter two distinct foci of ectopic thyroid tissue in a patient. Such a case also with subclinical hypothyroidism is reported here.

## CASE REPORT

**Figure 1:**
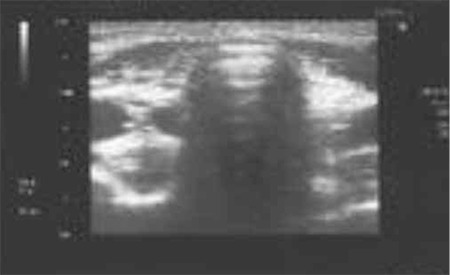
USG image of the neck. No visible thyroid tissue in neck USG

**Figure 2:**
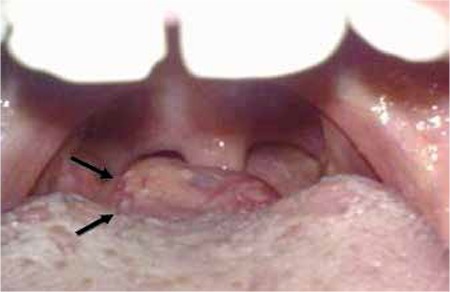
Lesion is seen in the right lateral base of the tongue with inspection(arrows)

**Figure 3:**
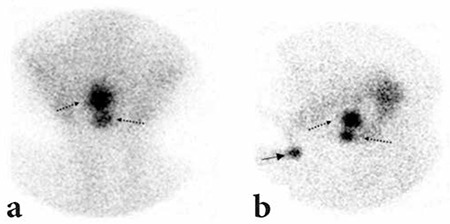
Tc-99m pertechnetate thyroid scintigraphy, pinhole image of theneck. Two foci of ectopic thyroid tissue are seen (broken arrows) in theanterior and lateral view, marker (arrow) is put on the sternal notch

A 10-year-old preadolescent girl incidentally presented with subclinical hypothyroidism. Ultrasonographic (USG) examination of the neck revealed the absence of a normally located thyroid gland (Figure 1). Physical examination revealed that a 2-3 cm mass at the tongue base (Figure 2). Thyroid function tests were in the normal range for free T3 and free T4 with elevated TSH of 32 IU/ml, compatible with subclinical hypothyroidism. A radionuclide thyroid scintigraphy with Technetium-99m (Tc-99m) pertechnetate showed two foci of intense uptake; one at the base of the tongue and the other in the infrahyoid region consistent with dual ectopic thyroid. There was no uptake of thyroid in the usual thyroid location in the neck (Figure 3). The patient had congenital dual ectopic thyroid and subclinical hypothyroidism, so she was started on replacement therapy.

**Literature Review and Discussion**

The thyroid gland develops in the floor of the primitive foregut between the first and second pharyngeal pouches from the endoderm. It descends in the anterior neck to the level of the trachea and is connected to the tongue base by the thyroglossal duct. The thyroglossal duct normally involutes or sometimes develops into a pyramidal lobe which is contiguous with the thyroid isthmus. Most congenital thyroid anomalies result from defective migration to the pretracheal position or incomplete obliteration of the thyroglossal duct. Ectopic thyroid tissue involves aberrant embryogenesis of the thyroid gland during its passage from the floor of the primitive foregut to the pretracheal position. Prevalence of ectopic thyroid gland is about 1 per 100.000-300.000 people and 1 per 4.000-8.000 patients with thyroid disease (1). 90% of undescended thyroid tissue occurs at the base of the tongue and is called lingual thyroid (3). Ectopic thyroid is a congenital anomaly that may present at any age and is usually detected during puberty or pregnancy because the increased demand for thyroid hormone elevates the TSH levels (4). Ectopic thyroid tissue may be also seen in the mediastinum, trachea, lung, porta hepatis, duodenum, esophagus, heart and breast (5).

Congenital hypothyroidism may be due to thyroid dysgenesis (ectopia, hypoplasia and agenesis) or occurs in eutopic glands. Subclinical hypothroidism is a clinic presantation of congenital hypothyroidism. In congenital hypothyroidism, thyroid scan with either Tc-99m perthecnetate or I-123 permits a precise characterization of the aetiology, which is important for genetic counselling and clinical management (6).

Lingual thyroid usually presents as a nodular mass at the base of the tongue in the midline. The lingual thyroid is four times more common in females than in males (7). Most patients with ectopic thyroid tissue are usually asymptomatic and thyroid hormone levels are normal. Major symptoms are dysphagia, dyspnea, foreign body sensation and bleeding (8). Hypothyroidism may be seen in up to 33% of cases (9). Clinically, any disease that affects normal thyroid glands can also affect ectopic thyroid tissue (5). Hyperplasia or inflammation may be also seen in lingual thyroid. Lingual thyroid should be evaluated with biopsy in cases of suspicious adenoma or carcinoma (10).

Dual ectopic thyroid tissue is very uncommon and has been reported in 43 cases in the search of the Pubmed in English literature. The age range of these patients was between 4 and 71 years and it was more common in females than males. The first lesion is usually seen in the lingual or sublingual region and the second lesion is subhyoid, infrahyoid or suprahyoid (1). Dual ectopic thyroid tissue with a normally located thyroid gland has been reported in only two cases in literature (3,11).

All the functional ectopic thyroid tissue can be determined by Tc-99m pertechnetate, I-131 or I-123 scintigraphy. To confirm diagnosis, scintigraphy is the most important method as it is a sensitive and specific imaging modality in the differential diagnosis of midline neck masses (1). Lateral views should be taken in addition to the anterior view to ensure that other ectopic thyroid tissue is not overlooked. The lateral views are helpful in the determination of anatomical position, and also useful in the detection of multiple ectopic thyroid tissues (12). Ectopic thyroid tissue may mimic thyroglossal duct cyst, but thyroid scintigraphy can differentiate these entities. Thyroglossal duct cysts do not contain enough ectopic follicular thyroid cells, so they are not detectable with scintigraphy (13,14). Uptake by salivary glands and nasal mucosa may cause physiological false positive scans, whereas meningioma, dacro-cystitis, prosthetic eye, sinusitis, dental pathologies and sialo-adenitis may show Tc-99m pertechnetate uptake and cause pathological false positive scans (15). For a decision of complete excision of lingual thyroid or transplantation of the ectopic tissue, thyroid scintigraphy is the method of the choice for the visualisation of normal thyroid tissue (11).

The normal thyroid gland is absent in about 70% of patients with lingual thyroid (2). Therefore, if surgical removal of the ectopic tissue is planned, the normal thyroid gland should be evaluated. In the case reported here there was no normal thyroid gland either on scintigraphy or in US examinations. Ectopic thyroid may be confused with oropharyngeal salivary gland. In our case the patient had subclinical hypothyroidism, there should be a thyroid tissue that produces thyroid hormone and so two foci of tissue are thought to be thyroid tissue.

There is no consensus on the optimal therapeutic strategy for ectopic thyroid tissue because of the rarity of this clinical entity. Surgical treatment is planned according to the age, size, local symptoms, functional thyroid status and complications. Complete surgical resection may be preferred if malignant transformation is suspected. Otherwise, follow up is suggested due to mass enlargement or complications in cases of asymptomatic and euthyroid status. For hypothyroid cases with mild symptoms, levothyroxine replacement may be effective and provides mass reduction (1). In the case reported here, the patient wasn’t operated and it was decided to follow-up the patient with levothyroxine replacement therapy due to elevated TSH unless any symptoms or complications occurred. In cases of subclinical hypothyroidism and nonvisualization of the thyroid gland by USG, thyroid scintigraphy is indicated for ectopic thyroid tissue.

**Conflicts of Interest**

There are no conflicts of interest.
